# PERK-mediated translational control is required for collagen secretion in chondrocytes

**DOI:** 10.1038/s41598-017-19052-9

**Published:** 2018-01-15

**Authors:** Satoshi Hisanaga, Masato Miyake, Shusuke Taniuchi, Miho Oyadomari, Masatoshi Morimoto, Ryosuke Sato, Jun Hirose, Hiroshi Mizuta, Seiichi Oyadomari

**Affiliations:** 1 Division of Molecular Biology, Institute for Genome Research, Institute of Advanced Medical Sciences, Tokushima University, Tokushima, 770-8503 Japan; 2 Department of Molecular Research, Diabetes Therapeutics and Research Center, Institute of Advanced Medical Sciences, Tokushima University, Tokushima, 770-8503 Japan; 3 Fujii Memorial Institute of Medical Sciences, Institute of Advanced Medical Sciences, Tokushima University, Tokushima, 770-8503 Japan; 4 Department of Orthopaedic Surgery, Faculty of Life Sciences, Kumamoto University, 1-1-1 Honjo, Chuo-ku, Kumamoto, 860-8556 Japan; 5 Department of Orthopaedic Surgery, Kumamoto University Hospital, 1-1-1 Honjo, Chuo-ku, Kumamoto, 860-8556 Japan; 6 Present Address: Department of Medical Informatics, Graduate School of Biomedical Sciences, Tokushima University, Tokushima, 770-8503 Japan

## Abstract

As chondrocytes are highly secretory and they experience a variety of stresses, physiological unfolded protein response (UPR) signalling is essential for extracellular matrix (ECM) secretion and chondrogenesis. In the three branches of the UPR pathway, PERK governs the translational attenuation and transcriptional upregulation of amino acid and redox metabolism and induction of apoptosis. It was previously demonstrated that a defect of the PERK branch of the UPR signalling pathway causes the accumulation of unfolded proteins, leading to cell death without perturbing endoplasmic reticulum (ER)-to-Golgi transport in pancreatic β cells. However, little is known about the role of PERK in chondrocytes. In this study, we found that PERK signalling is activated in chondrocytes, and inhibition of PERK reduces collagen secretion despite causing excessive collagen synthesis in the ER. *Perk*^*−/−*^ mice displayed reduced collagen in articular cartilage but no differences in chondrocyte proliferation or apoptosis compared to the findings in wild-type mice. PERK inhibition increases misfolded protein levels in the ER, which largely hinder ER-to-Golgi transport. These results suggest that the translational control mediated by PERK is a critical determinant of ECM secretion in chondrocytes.

## Introduction

Cartilage is characterised by a structurally arranged extracellular matrix (ECM) composed of collagen and non-collagenous proteins such as proteoglycans^1,2^. The chondrocyte is the sole resident cell type in articular cartilage, and this highly specialised cell plays a crucial role in ECM maintenance. As articular cartilage is avascular, chondrocytes exist at low oxygen tension and under limited nutrient conditions. For example, oxygen tension ranges from 1% in the deep layers of articular cartilage to approximately 6% at the joint surface and less than 7% in synovial fluid^3,4^. The glucose concentration surrounding chondrocytes within articular cartilage has been estimated to be 1 mM or lower, versus 4–6 mM in synovial fluid^5^. ECM production in articular chondrocytes is affected by its microenvironment, which, in return, affects the mechanical resilience of cartilage. Reduced ECM content is linked to the progression of degenerative joint diseases such as osteoarthritis (OA).

Secreted and membrane proteins are folded and assembled in the endoplasmic reticulum (ER) before transport to the extracellular space or other cellular compartments. Poorly folded proteins are retained in the ER and targeted for degradation, and this ER protein quality control mechanism can be overwhelmed by various insults, such as hypoxia or low nutrients, resulting in ER stress. To alleviate ER stress, cells activate the so-called unfolded protein response (UPR). Under adaptive conditions, the UPR induces attenuation of protein synthesis to reduce the ER load via PERK signalling, inducing ER chaperones to assist protein folding mainly via ATF6 signalling and activating ER-associated degradation to eliminate misfolded proteins mainly via IRE1 signalling^6,7^. However, when the stress exceeds the capacity of the ER homeostatic machinery, cells undergo apoptosis^8^.

As chondrocytes are highly secretory and they experience a variety of stresses, physiological UPR signalling appears essential for ECM secretion and chondrogenesis^9–11^. The importance of each UPR signalling branch for ECM secretion and chondrogenesis is apparent from gene targeting studies^12^. Activation of IRE1 pathway such as IRE1 phosphorylation and IRE1’s downstream target XBP1 splicing was observed in differentiating chondrocytes^12^. Cartilage-specific XBP1 knockout mice displayed a chondrodysplasia involving dysregulated chondrocyte proliferation and growth plate hypertrophic zone shortening, indicating roles of XBP1 in regulating chondrocyte proliferation and cartilage maturation^13^. Although ATF6 knockout mice have no defect on skeletal development^14^, ablation of *Creb3l2*, a ATF6 homology preferentially expressed in chondrocytes exhibited a short limbs and reduced cartilage matrix^15^. The chondrocytes of *Creb3l2* knockout mice displayed a delayed expression of differentiation markers and sever ER stress with the accumulation of ECM aggregates in the ER, indicating that *Creb3l2* is critical for chondrocyte differentiation and ECM transport from the ER-to-Golgi^16^. PERK knockout mice are defective in both membranous and endochondral ossification and growth retardation^17,18^. Mice with cartilage-specific knockout of ATF4, which is a downstream transcription factor of PERK signalling, also displayed a short stature and delayed endochondral ossification^19^. Furthermore, PERK-deficient osteoblasts showed impaired osteoblast differentiation and compromised trafficking and secretion of type I collagen and abnormal retention of procollagen I in the ER^20^. However, the contribution of PERK to chondrocyte differentiation and ECM secretion has not been extensively investigated.

As evidenced by the severe chondrodysplasia of these UPR-defective mice, UPR signalling is essential for maintaining chondrocyte homeostasis. We previously reported that ER stress is induced in chondrocytes from OA mouse models^21^ and human patients^22^. We also uncovered that reducing ER stress-mediated apoptosis mitigates OA progression in an OA mouse model^23^. Although the role of UPR signalling on chondrocyte death has been investigated, it is unknown whether the UPR is involved in decreased ECM secretion in the presence of cartilage disorders. In this study, we demonstrate that inhibition of PERK decreases collagen secretion without affecting cell proliferation and death. Our finding indicates that the translational control regulated by PERK is required for collagen secretion in chondrocytes.

## Results

### Activation of PERK signalling occurs during chondrogenic differentiation in ATDC5 cells

**Figure 1:**
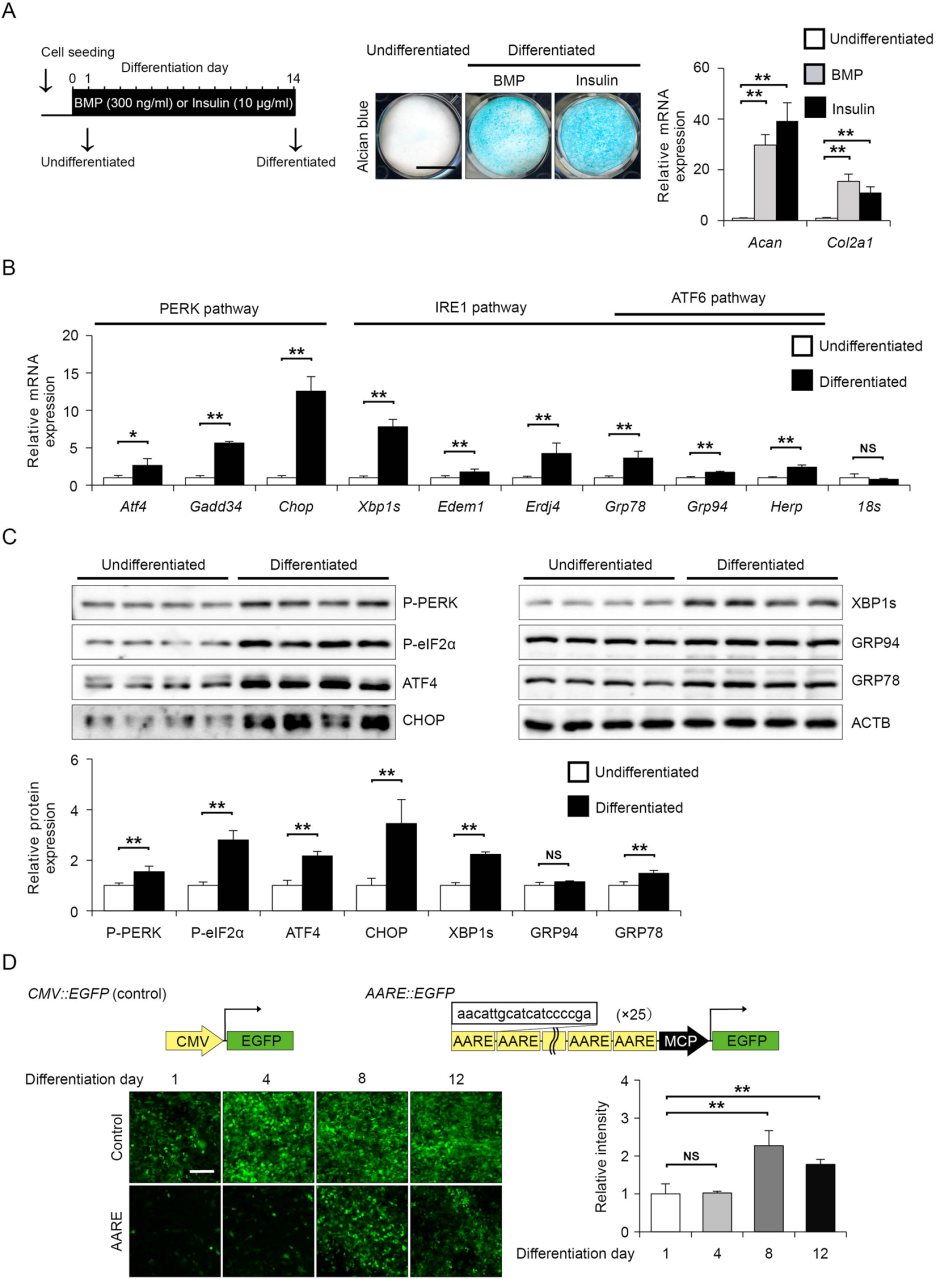
Activation of the PERK pathway upon the chondrogenic differentiation of ATDC5 cells in response to insulin. (**A**) Representative alcian blue staining and RT-qPCR analysis of aggrecan (*Acan*), type 2A1 collagen (*Col2a1*) expression in ATDC5 cells at differentiation days 1 (undifferentiated) and 14 (differentiated). ATDC5 cells were induced to differentiate into chondrocytes with 10 μg/ml insulin or 300 ng/ml BMP. Data are presented as the mean fold change ± SD in differentiated cells versus that in undifferentiated cells (*n* = 4 technical replicates, **P < 0.01). Scale bar, 10 mm. (**B**) RT-qPCR analysis of *Atf4, Gadd34, Chop*, *Xbp1s*, *Edem*, *Erdj4*, *Grp78*, *Grp94*, *Herp* and 18 *s* expression in undifferentiated or differentiated ATDC5 cells. The mRNA levels were normalised to those of β-actin. Data are presented as the mean fold change ± SD in differentiated cells versus that in undifferentiated cells (*n* = 4 technical replicates, *P < 0.05, **P < 0.01). (**C**) Representative immunoblots and quantification of phosphorylated PERK, phosphorylated eIF2α, ATF4, CHOP, XBP1s, GRP94, GRP78 and ACTB in undifferentiated or differentiated ATDC5 cells. The protein levels were normalised to those of ACTB. Data are presented as the mean fold change ± SD in differentiated cells versus that in undifferentiated cells (*n* = 4 technical replicates, **P < 0.01). (**D**) Representative fluorescence micrographs of *CMV::EGFP* and *AARE::EGFP* ATF4 reporter ATDC5 cells on the indicated day after insulin stimulation. The fluorescence intensity ratio of target (*AARE::EGFP*) to control (*CMV::EGFP*) was determined and normalized to undifferentiated cells. The schema represents control and ATF4 activity GFP reporter constructs containing 25 tandem amino acid response elements (AAREs) (*n* = 4 technical replicates, **P < 0.01, NS = not significant). Scale bar, 200 μm.

As chondrocytes secrete abundant ECM proteins, ER stress has been implicated in chondrocyte proliferation, differentiation and hypertrophy^16^. First, we verified whether the UPR is activated during the chondrogenic differentiation of the mouse embryonal carcinoma-derived cell line ATDC5. ATDC5 provides an excellent *in vitro* model that exhibits chondrogenic differentiation by adding BMP2 or insulin^24^. In presence of BMP2 or insulin for 14 days, undifferentiated ATDC5 cells converted into chondrogenic cells which were strongly stained with alcian blue, and exhibited strong induction of type 2A collagen (*Col2a1*) and aggrecan (*Acan*) mRNA (Fig. 1A). As insulin induced chondrogenic conversion as same as BMP2, we used insulin for chondrogenic differentiation in subsequent experiments. Next, we examined whether UPR downstream target genes were induced in the differentiated ATDC5 cells via RT-qPCR analysis. The expression of PERK pathway downstream target genes such as *Atf4*, *Gadd34* and *Chop* was increased by 2.7-, 5.6- and 12.6-fold, respectively, in the differentiated ATDC5 cells (Fig. 1B). Similar to PERK pathway activation, the expression of IRE1 or ATF6 pathway downstream genes such as the spliced form of *Xbp1* (*Xbp1s*), *Edem*, *Erdj4*, *Grp78*, *Grp94* and *Herp* was also increased by 7.8-, 1.8-, 4.2-, 3.6-, 1.7- and 2.4-fold, respectively, in the differentiated ATDC5 cells (Fig. 1B). Whereas, expression of housekeeping gene 18 S rRNA was not affected during chondrogenic differentiation. In addition to mRNA levels, the protein levels of UPR markers were also determined. The levels of phosphorylated PERK and eIF2α were increased by 1.6- and 2.8-fold in the differentiated ATDC5 cells, respectively, and the expression of ATF4 and CHOP was also increased by 2.2- and 3.5-fold, respectively, indicating PERK pathway activation (Fig. 1C). The expression of XBP1s and GRP78 was increased by 2.2- and 1.5-fold, respectively, in the differentiated ATDC5 cells (Fig. 1C). Thus, activation of the IRE1 and ATF6 pathways was observed, but the PERK pathway appeared to be more strongly activated in the differentiated ATDC5 cells than the other two branches of the UPR pathway. PERK-dependent UPR target genes are largely known to be regulated by ATF4^25,26^. Therefore, we monitored ATF4 activation using a reporter in which GFP was bound to the amino acid response element (AARE), which is the response sequence of ATF4. The GFP intensity of the CMV promoter-driven reporter remained unchanged, but the GFP intensity of the AARE reporter was increased as ATDC5 cells differentiated into chondrocytes (Fig. 1D). These results demonstrated that chondrocyte differentiation in ATDC5 cells activates the three branches of the UPR, among which the PERK pathway was most significantly activated.

### Loss of PERK signalling increases intracellular COL2A1 levels but decreases extracellular COL2A1 levels *in vitro*

**Figure 2:**
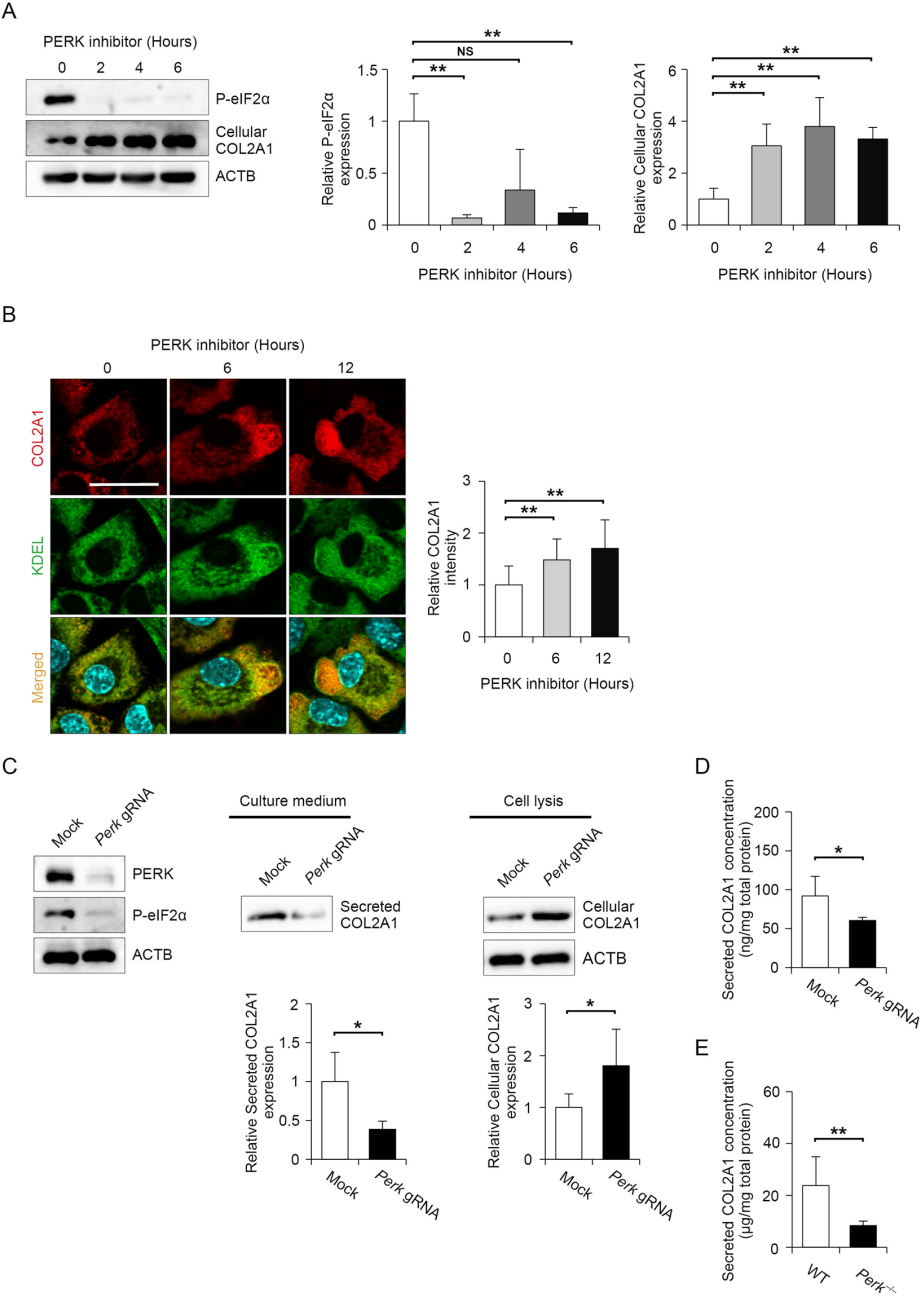
Type 2 A collagen (COL2A1) secretion was diminished by PERK inhibition, whereas intercellular COL2A1 expression was increased. (**A**) Representative immunoblots of phosphorylated eIF2α, cellular COL2A1 and ACTB in mouse primary chondrocyte treated with 2 μM PERK inhibitor for the indicated times. The protein levels were normalised to those of ACTB. Data are presented as the mean fold change ± SD compared to that in untreated cells (*n* = 4 technical replicates, **P < 0.01, NS = not significant). (**B**) Representative images of immunostaining for COL2A1 and KDEL (ER localization marker) in mouse primary chondrocytes treated with 2 μM PERK inhibitor for the indicated times. Relative fluorescence intensity was quantified in comparison to that of untreated cells and each group data consisted of images of 10 cells (*n* = 10 technical replicates, *P < 0.05). Scale bar, 200 μm. (**C**) Representative immunoblots of PERK, ACTB, phosphorylated eIF2α and secreted and cellular COL2A1 in collagen-secreting rat chondrosarcoma (RCS) cells stably expressing Cas9 and mock gRNA or three *Perk*-specific gRNAs. Data are presented as the mean fold change ± SD compared to that in mock gRNA-expressing cells (*n* = 4 technical replicates, **P < 0.01). (**D**) Quantitative assessment of secreted COL2A1 in collagen-secreting RCS cells stably expressing Cas9 and mock gRNA or three *Perk*-specific gRNAs. Values were normalised by the total protein amount of the cells and shown as the mean ± SD (*n* = 4 technical replicates, *P < 0.05). (**E**) Quantitative assessment of secreted COL2A1 in primary chondrocytes derived from wild-type or *Perk*^***−/−***^ mice at 18.5 days postcoitum (dpc). Values were normalised by the total protein amount of the cells and shown as the mean ± SD (*n* = 5 technical replicates, *P < 0.05).

Analysis using PERK-deficient mice illustrated that PERK is essential for maintaining the functions of secreted pancreatic β cells and osteoblasts^18,27,28^, but its role in chondrocytes has not been analysed. To detect whether the loss of PERK activity can affect the function of chondrocytes, we first analysed the effect of the specific PERK inhibitor GSK2606414 on the synthesis of COL2A1, which is a major ECM component secreted by chondrocytes. Exposure to 2 μM GSK2606414 decreased the basal phosphorylation level of eIF2α to approximately 10% of control levels in primary chondrocytes isolated from mice (Fig. 2A). Intracellular COL2A1 expression was inversely correlated with eIF2α phosphorylation after PERK inhibition (Fig. 2A). To confirm the increased intracellular COL2A1 expression, we visualized PERK inhibitor-treated cells by staining COL2A1 and KDEL retrieval sequence as an ER localization marker. Immunocytochemistry revealed that COL2A1 expression gradually increased after 6 and 12 h of PERK inhibitor treatment, and the protein appeared to accumulate in the vacuole-like ER (Fig. 2B). To confirm the effect of PERK inhibition, we genetically disrupted the Perk gene using the CRISPR/Cas9 system with multiple Perk-specific gRNAs in collagen-secreting rat chondrosarcoma (RCS) cells. The expression of PERK and phosphorylated eIF2α was almost completely suppressed in Perk-mutated RCS cells (Fig. 2C). Consistent with the results of PERK inhibitor administration, intracellular COL2A1 expression was increased by nearly 2-fold, whereas extracellular COL2A1 secretion was decreased to 0.4-fold of control levels (Fig. 2C). Consistent with immunoblot data, ELISA analysis demonstrated that extracellular COL2A1 secretion decreased to 0.6-fold of the control levels in *Perk*-deleted RCS cells (Fig. 2D). To verify the observation from the *Perk*-deleted RCS cells, we examined extracellular COL2A1 secretion from the primary chondrocytes, which were isolated from wild-type or *Perk*^*−/−*^ mice. Consistent with the RCS cell study, extracellular COL2A1 secretion was decreased to 0.3-fold of control levels in the *Perk*^*−/−*^ primary chondrocytes compared to the wild-type primary chondrocytes (Fig. 2E). Taken together, these results demonstrate that decreased PERK activity results in increased intracellular COL2A1 expression and decreased extracellular COL2A1 expression in chondrocytes.

### Loss of PERK signalling decreases extracellular COL2A1 expression without reducing proliferation or increasing apoptosis in chondrocytes

**Figure 3:**
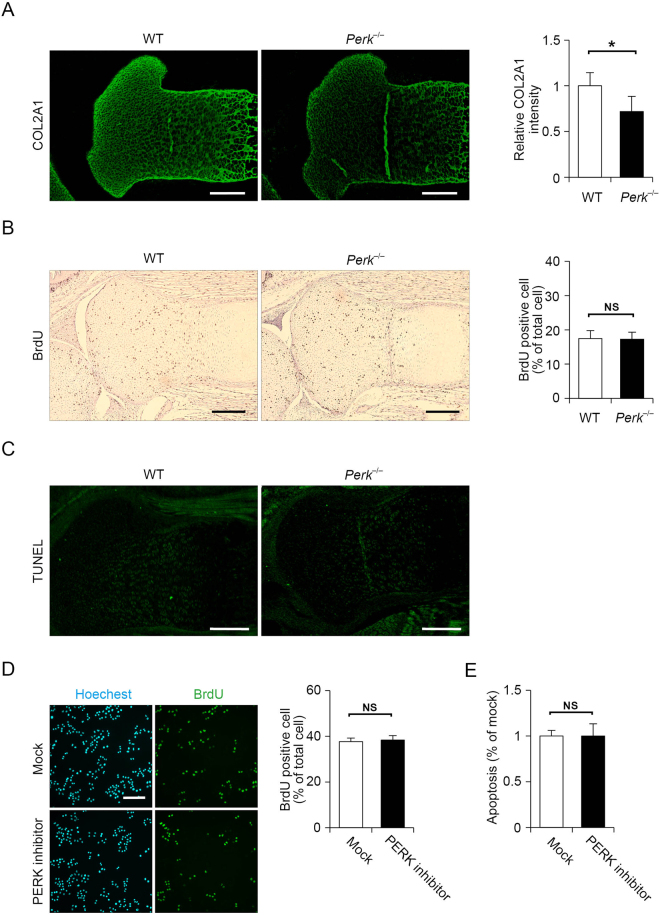
Type 2A collagen (COL2A1) expression was decreased in *Perk*^*−/−*^ mice without a reduction of proliferation or increase in apoptosis in chondrocytes. (**A**) Representative fluorescence micrographs of immunohistochemistry for COL2A1 of tibial sections from wild-type or *Perk*^***−/−***^ mice at 18.5 dpc. Scale bar, 200 μm. Relative fluorescence intensity was presented as the mean fold change ± SD versus that of wild-type mice (*n* = 6 technical replicates, *P < 0.05). (**B**) Representative micrographs of immunohistochemistry for bromodeoxyuridine (BrdU) in tibial sections from wild-type or *Perk*^***−/−***^ mice at 18.5 dpc. The sections were counterstained using hematoxylin. Scale bar, 200 μm. Ratio of BrdU-positive cells divided by the total number of cells was expressed as the mean ± SD (*n* = 5 technical replicates, NS = not significant). (**C**) Representative micrographs of TUNEL staining of tibial sections from wild-type or *Perk*^*−/−*^ mice at 18.5 dpc. Scale bar, 200 μm. (**D**) Representative micrographs of immunohistochemistry for BrdU of rat chondrosarcoma cells treated with mock inhibitor or 2 μM PERK inhibitor for 12 h. The cells were counterstained using Hoechest 33258. Ratio of BrdU-positive cells divided by the total number of cells was expressed as the mean ± SD (*n* = 4 technical replicates, NS = not significant). Scale bar, 200 μm. (**E**) Quantitative assessment of apoptotic DNA fragmentation via nucleosomal DNA ELISA measuring the relative increase in primary chondrocytes treated with mock inhibitor or 2 μM PERK inhibitor for 24 h (*n* = 4 technical replicates, NS = not significant). Values were normalised by the total protein amount of the cells and shown as the mean ± SD.

To examine whether decreases in COL2A1 secretion in PERK-defective cultured chondrocytes are also observed in articular cartilage, we analysed *Perk*^*−/−*^ mice. It is known that joint formation occurs at day 18.5 of embryogenesis in wild-type mice. Hence, we performed immunostaining for COL2A1 in the growth plate of the tibia in wild-type and *Perk*^*−/−*^ mice at 18.5 days postcoitum (dpc). COL2A1 expression was significantly decreased in the growth plate of *Perk*^*−/−*^ mice compared with that in wild-type mice (Fig. 3A). Earlier studies on *Perk*^*−/−*^ mice revealed that PERK deficiency leads to reduced proliferation and increased apoptosis in insulin-secreting pancreatic β cells^17,27,28^. However, little is known regarding the impact of PERK deficiency in chondrocytes. Therefore, we investigated the effect of PERK deficiency on proliferation and apoptosis in chondrocytes using *Perk*^*−/−*^ mice. To examine proliferation in Perk-deficient limbs, we measured bromodeoxyuridine (BrdU) incorporation into proliferating chondrocytes at 18.5 dpc. No significant difference was noted concerning the number of proliferating cells incorporating BrdU between wild-type and *Perk*^*−/−*^ mice (Fig. 3B). Unlike pancreatic β cells, our result suggests that PERK deficiency does not affect chondrocyte proliferation. Next, we assessed chondrocyte apoptosis in the growth plate of the tibia at 18.5 dpc by TUNEL staining. We did not detect any TUNEL-positive cells in either wild-type or *Perk*^*−/−*^ mice, suggesting that PERK deficiency does not induce apoptosis (Fig. 3C). Proliferation and apoptosis of chondrocytes may be regulated differently depending on their developmental stages. Therefore, we determined COL2A1 expression, BrdU incorporation, and TUNEL-positive apoptosis in the growth plate of the tibia at 16.5 dpc. Consistent with the observation at 18.5 dpc, reduced COL2A1 expression in *Perk*^*−/−*^ mice, but no significant difference on chondrocyte proliferation and apoptosis between wild-type and *Perk*^*−/−*^ mice were observed at 16.5 dpc (Supplemental Fig. 1). Because of our technical limitation, we could not examine that at the earlier developmental stage than 16.5 dpc, but at least our observation is consistent with two different developmental stages. As cells in tissue are heterogeneous and their proliferation and apoptosis are not synchronised, it is possible to underestimate changes of proliferation or apoptosis under PERK-deficient conditions. Thus, we verified the effects of PERK deficiency on proliferation and apoptosis in cultured cells using a PERK inhibitor. Consistent with the results in *Perk*^*−/−*^ mice, RCS cells treated with a PERK inhibitor exhibited no differences in cell proliferation and apoptosis from mock-treated cells (Fig. 3D,E). Thus, the data indicate that decreases in extracellular COL2A1 expression associated with the loss of PERK signalling do not lead to reduced proliferation or increased apoptosis in chondrocytes.

### Inhibition of PERK disturbs ER protein folding homeostasis, which inhibits COL2A1 transport from the ER-to-Golgi in chondrocytes

**Figure 4:**
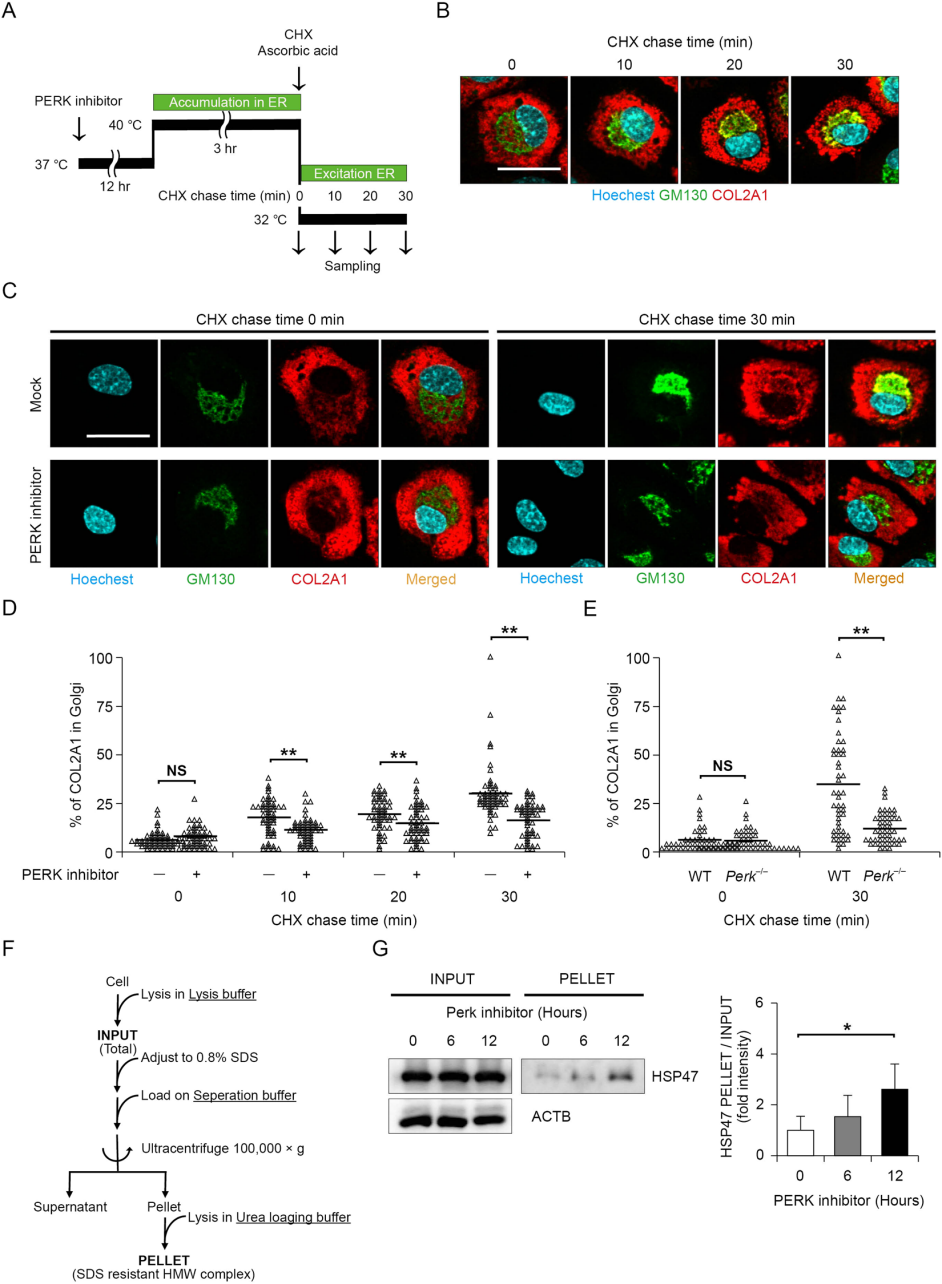
PERK inhibition led to accumulation of the high-molecular-weight type 2 A collagen (COL2A1), resulting in blockade of endoplasmic reticulum (ER)-to-Golgi transport. (**A**) Protocol for the procollagen transport assay. Mouse primary chondrocytes were treated with 2 μM PERK inhibitor for 12 h and exposed to a temperature of 40 °C for 3 h (accumulation in ER), followed by a temperature shift to 32 °C combined with exposure to 100 μg/ml cycloheximide and 50 μg/ml ascorbic acid for the indicated times (exit from ER). Procollagen transport was visualised by immunostaining. (**B**) Representative micrographs of immunostaining for Hoechest 33258, GM130 and COL2A1 in mouse primary chondrocytes after ER block release in the procollagen transport assay. Scale bar, 50 μm. (**C**) Representative micrographs of immunostaining for Hoechest 33258, GM130 and COL2A1 in mouse primary chondrocytes before and after ER block release in the procollagen transport assay. Mouse primary chondrocytes were pretreated with mock inhibitor or 2 μM PERK inhibitor for 12 h. Scale bar, 50 μm. (**D**) Quantification of transported COL2A1 in mock inhibitor- and PERK inhibitor-treated mouse primary chondrocytes. A scatter plot comparing mock inhibitor- and PERK inhibitor-treated mouse primary chondrocytes regarding the ratio of Golgi-localised COL2A1 to total COL2A1 is shown and each group data consisted of images of 10 cells (*n* = 5 technical replicates, **P < 0.01). (**E**) Quantification of transported COL2A1 in primary chondrocytes derived from wild-type or *Perk*^***−/−***^ mice at 18.5 dpc and each group data consisted of images of 10 cells (*n* = 5 technical replicates, **P < 0.01). (**F**) Protocol for high-molecular-weight (HMW) detergent-resistant complex separation to measure COL2A1 folding. Mouse primary chondrocytes treated with 2 μM PERK inhibitor for the indicated times were lysed in lysis buffer, adjusted to 0.8% SDS, loaded on separation buffer and then ultracentrifuged at 100,000 × *g* for 45 min. The HMW detergent-resistant complex containing the collagen-specific chaperon HSP47 represents unfolded or misfolded COL2A1. (**G**) Representative immunoblots and quantification of HSP47 in SDS-resistant HMW complexes (PELLET) and total cell lysate (INPUT) from mouse primary chondrocytes treated with 2 μM PERK inhibitor for the indicated times. The protein levels of HSP47 in PELLET were normalised to the total HSP47 levels in INPUT and shown as the mean ± SD (*n* = 5 technical replicates, *P < 0.05).

Because PERK plays a role in attenuating translation in response to ER stress, inhibiting PERK function leads to excessive protein synthesis in the ER. In chondrocytes, ER overload caused by PERK dysfunction remarkably decreases COL2A1 secretion, and therefore, we sought to clarify the molecular mechanism. To investigate the mechanism by which COL2A1 secretion is suppressed, we examined changes in COL2A1 transport from the ER-to-Golgi using a PERK inhibitor. Several groups reported a procedure to synchronise ER-to-Golgi collagen transport^29,30^. Concretely, COL2A1 folding can be blocked at 40 °C, and unfolded or misfolded COL2A1 accumulates in the ER. COL2A1 accumulation can be rapidly reversed by reducing the temperature to 32 °C and adding ascorbic acid to the culture medium. To monitor COL2A1 transport precisely, we performed a cycloheximide (CHX) chase combined with temperature-induced ER block release of COL2A1 (Fig. 4A). Immunostaining of mouse primary chondrocytes displayed CHX chase time-dependent colocalization (yellow) of COL2A1 (red) and the Golgi apparatus marker GM130 (green), demonstrating that ER-to-Golgi COL2A1 transport was nicely monitored using a CHX chase (Fig. 4B). The COL2A1 retention in the ER was reversed in the mock-treated mouse primary chondrocytes, but not in mouse primary chondrocytes treated with the PERK inhibitor at the 30 mins CHX chase point after shifting the temperature to 32 °C and adding ascorbic acid (Fig. 4C,D). Consistent with the results in mouse primary chondrocytes treated with a PERK inhibitor, primary chondrocytes from *Perk*^*−/−*^ mice exhibited the COL2A1 retention in the ER at the 30 mins CHX chase point after shifting the temperature to 32 °C and adding ascorbic acid (Fig. 4E). These results suggest that the recovery of ER-to-Golgi transport of COL2A was largely delayed in PERK inhibition. We hypothesised that protein synthesis exceeding the folding capacity of the ER might compromise the folding of all newly synthesised proteins in the ER and consequently delay ER-to-Golgi protein transport. To test the hypothesis, we evaluated the amount of misfolded proteins in the ER when chondrocytes were treated with a PERK inhibitor. Previously, we developed a method to estimate the amount of misfolded protein in the ER based on the level of the SDS-resistant high-molecular-weight (HMW) HSP47-containing complex in fibroblasts^31^ (Fig. 4F). In mouse primary chondrocytes, HSP47 levels in the PELLET fraction corresponding to misfolded protein in the ER were increased by PERK inhibitor treatment, whereas the levels of HSP47 in the INPUT fraction corresponding to total cellular HSP47 was unchanged (Fig. 4G). Altogether, these results support the concept that PERK inhibition disturbs ER protein folding homeostasis, which inhibits ER-to-Golgi COL2A1 transport in chondrocytes.

## Discussion

**Figure 5:**
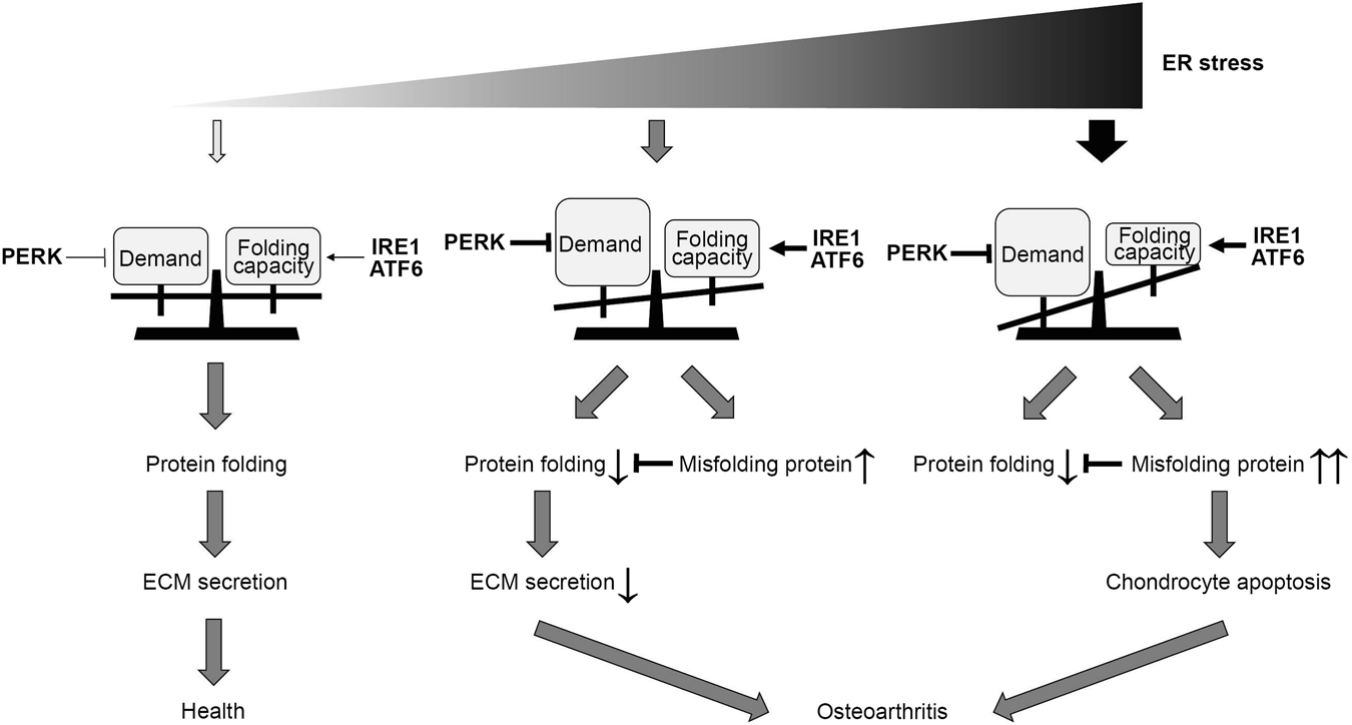
Proposed schematic model of the role of PERK in chondrocyte pathophysiology. When the endoplasmic reticulum (ER) folding capacity controlled by the unfolded protein response is balanced with the demand for extracellular matrix (ECM) secretion, ECM is efficiently secreted, and the health of chondrocytes can be maintained. However, when the demand for ECM secretion exceeds the folding capacity, ECM secretion is decreased, and chondrocytes undergo apoptosis. The translational control mediated by PERK is a critical determinant of this chondrocyte pathophysiology.

OA is the most common chronic joint disease in the elderly, and it is characterised by the progressive destruction of articular cartilage, resulting in severe locomotor disability. Cartilage destruction is known to be caused by ECM degeneration and chondrocyte death. The mechanism of enhanced ECM degradation and increased chondrocyte apoptosis has been extensively studied, but little research has focused on reductions in ECM regeneration. Recent experimental data indicated that ER stress and the UPR contribute to chondrocyte apoptosis in cartilage pathophysiology^32,33^, but their contribution to ECM secretion is not completely understood. In this study, we found that PERK signalling is activated in chondrocytes, and inhibition of PERK reduces collagen secretion despite inducing excessive collagen synthesis in the ER. Indeed, *Perk*^*−/−*^ mice exhibited reduced collagen content in articular cartilage but no differences in chondrocyte proliferation or apoptosis compared to the findings in wild-type mice. PERK inhibition increases misfolded protein levels in the ER and hinders ER-to-Golgi protein transport. Misfolded proteins work in a dominant-negative manner. These results suggest that the translational control mediated by PERK is a critical determinant of chondrocyte pathophysiology (Fig. 5).

Among ECM proteins, collagen is of particular relevance to chondrogenesis and endochondral bone formation. Collagen undergoes translational modifications accompanied by its folding. For instance, hydroxylation of specific proline or lysine residues, *O*-glycosylation of specific hydroxylysine residues, and *N*-glycosylation of specific asparagine residues begin in the ER. Folding of the triple helix occurs in the ER together with the assembly of C-propeptides and formation of disulfide bonds. Consequently, disruption of collagen biosynthesis and assembly in the ER can occur at several steps, resulting in ER stress. The most significant examples were uncovered in studies of mutations in collagen-encoding genes^34^. Mutations in Col2A1 were reported to activate the UPR, leading to chondrocyte apoptosis and contributing to early OA^35,36^. In this study, we found that increased production of misfolded COL2A1 exerted a dominant effect by reducing collagen secretion. In addition, mutants of collagens and other ECM proteins such as cartilage oligomeric matrix protein (COMP), aggrecan (ACAN) and matrilin-3 have been demonstrated to trigger ER stress and activate the UPR^37–39^. Thus, we speculated that the misfolded ECM proteins in chondrocytes are poorly secreted and largely retained in the ER, presumably via ER-associated degradation. As shown in Fig. 5, misfolded proteins cause secretion disorders, and further accumulation of misfolded proteins is believed to ultimately result in cell death. The diverging point of secretion disorders and cell death varies among cell types. In pancreatic β cells, PERK deletion did not disrupt ER-to-Golgi transport, but it increased cell death^28^. However, in chondrocytes, PERK deletion inhibited ER-to-Golgi transport without inducing cell death. This cell-type-specific difference could be explained by cell-type-specific ER environments or ER client proteins. Interestingly, Li *et al*. demonstrated that silencing *PERK* reduced COL2A1 expression in human oseoarthiritic chondrocytes^40^, consisted with our observation of reduce COL2A1 secretion caused by the *Perk* deletion and the PERK activity inhibition. Li *et al*. also showed that silencing *PERK* resulted in an increase of COL1A1 expression in human osteoarthritic chondrocytes^40^. It is known that COL2A1 is reduced, while COL1A1 is increased in the course of the progression of human OA^41^. Therefore, PERK might differentially regulate collagen secretion depending on the type of collagen or on the different stages of OA and could be an important determinant of OA progression. In contrast, the complexity of protein folding and amount of newly synthesised proteins appear to be important determinants of PERK activation. Further investigations on the nature of PERK activaton and collagen-type-specific protein synthesis are required.

Concerning the regulation of ER protein homeostasis, mice lacking the essential autophagy-related gene 7 (*Atg7*) in chondrocytes displayed accumulation of COL2 in the ER and lower levels of COL2 in cartilage compared to the findings in wild-type mice^29^, suggesting that autophagy influences collagen secretion by controlling ER homeostasis. The reduced COL2 secretion phenotype of *Atg7*^*−/−*^ mice due to inhibition of ER-to-Golgi transport resembles that in cartilage from *Perk*^*−/−*^ mice. Conversely, deletion of P58^IPK^, the cellular inhibitor of PKR and PERK, has been shown to cause bone changes and joint degeneration in mice^42^. Although, the the extent of ER stress and level of protein translation was not determined, upregulated phosphorylation of PKR and PERK was demonstrated in P58^IPK^–null mice^42^. It is not easy to reconcile why chondrodysplastic phenotypes were observed in both PERK-deleted and PERK-hyperactivated conditions. One explanation may suggest that PERK has another function besides collagen synthesis regulation, such as chondrocyte differentiation. Using a mouse model expressing misfolding collagen and lacking IRE1 pathway in chondrocytes, Cameron *et al*. demonstrate that disruption of C/EBPβ, a regulator of chondrocyte differentiation, by CHOP, a transcription factor downstream of PERK that inhibits C/EBP proteins, and down-regulation of C/EBPβ transcriptional co-factors, GADD45β and RUNX2^43^. These findings suggest that excess activation of PERK may contribute chondrodysplastic phenotype by dysregulation of chondrocyte dedifferentiation. Taken together, proper activation of PERK is crucial to maintain chondrocyte homeostasis. Our findings suggest that PERK may be a potential target for treating OA.

## Methods

### Mice

*Perk*^*−/−*^ mice were previously described^44^. The experimental protocols involving animals were approved by the Animal Research Committee of Tokushima University, and all experiments were performed in accordance with the appropriate institutional guidelines.

### Cell culture, transfection and transduction

ATDC5 and RCS cells were maintained in DMEM/F-12 (Nacalai Tesque) supplemented with 5% fetal bovine serum (FBS, Gibco) and DMEM (Nacalai Tesque) supplemented with 10% FBS, respectively. For chondrocyte differentiation, confluent ATDC5 cells were cultured in maintenance medium supplemented with 10 μg/ml bovine insulin (Wako) which is refered to differentiation medium^45^. The culture medium was replaced daily. In some experiments, the cells were cultured in maintenance medium supplemented with 300 ng/ml human recombinant BMP-2(Miltenyi Biotec) instead of insulin. Primary chondrocytes were isolated from the limbs of 18.5 dpc embryonic or newborn mice as previously reported^46^. To inhibit the enzymatic activity of PERK, primary chondrocytes or RCS cells were treated with the highly specific PERK inhibitor GSK2606414 (Calbiochem)^47^. Transfection was performed using polyethylenimine (Polysciences) as described previously^48^. Lentiviral transduction was performed using a previously published protocol with modifications^49^.

### Genome editing using the multiplex CRISPR/Cas9 assembly system

The specific gRNA sequences were selected using the CRISPR design tool (http://crispr.mit.edu/) and cloned into the pX330A-1 × 3 multiplex gRNA assembly vector (Addgene plasmid #58767)^50^ using the Golden Gate cloning method. Then, the multiplex gRNA cassette was replaced with the lentiviral vector LentiGuide-Puro (Addgene plasmid #52963). Stable expression of Cas9 and gRNAs was established via puromycin or blasticidin resistance using LentiCas9-Blast (Addgene plasmid #529632) following Zhang *et al*.’s protocol^51^.

### RT-PCR analysis

Total RNA was subjected to RT using RiverTra Ace qPCR RT Master Mix with a gDNA Remover kit (TOYOBO Life Science) according to the manufacturer’s protocol. RT-PCR was performed using Power SYBR Green PCR Master Mix (Applied Biosystems). Real-time RT-PCR was performed using the StepOnePlus Real-Time PCR System (Applied Biosystems). The primer information is summarised in Table 1.

**Table 1:** Primer list.

No	Oligo name	Gene	Sequence	Purpose
1	mPerk.SP1	*Perk*	aaggaccctatcctcctgctgcac	Genotyping
2	mPerk.SP2	*Perk*	gctaccggtggatgtggaatgtg	Genotyping
3	mPerk.AP1	*Perk*	cggagacagtacaagcgcagatga	Genotyping
4	mCol2A1.SP1	*Col2a1*	gggtctcctgcctcctcctgc	RT-PCR
5	mCol2A1.AP1	*Col2a1*	tcctttctgcccctttggccctaattttcg	RT-PCR
6	mAcan.SP1	*Acan*	gtcgggagcagcagtcacatctgagcag	RT-PCR
7	mAcan.AP1	*Acan*	ccattcgcctctctcatgccagatc	RT-PCR
8	mActB.SP1	*Actb*	ccgccctaggcaccagggtg	RT-qPCR
9	mActB.AP1	*Actb*	ggctggggtgttgaaggtctcaaa	RT-qPCR
10	mAtf4.SP1	*Atf4*	atgatggcttggccagtg	RT-qPCR
11	mAtf4.AP1	*Atf4*	ccattttctccaacatccaatc	RT-qPCR
12	mGadd34.SP1	*Gadd34*	tcctctaaaagctcggaaggt	RT-qPCR
13	mGadd34.AP1	*Gadd34*	caaagcggcttcgatctc	RT-qPCR
14	mChop.SP1	*Chop*	ccaccacacctgaaagcag	RT-qPCR
15	mChop.AP1	*Chop*	tcctcataccaggcttcca	RT-qPCR
16	mXbp1s.SP1	*Xbp1*	gctgagtccgaatcaggtgcagg	RT-qPCR
17	mXbp1s.AP1	*Xbp1*	tccttctgggtagacctctgggag	RT-qPCR
18	mEdem1.SP1	*Edem1*	ggtcttcgaagctacgataagg	RT-qPCR
19	mEdem1.AP1	*Edem1*	gggctgtttggaatcagttatta	RT-qPCR
20	mErdj4.SP1	*Erdj4*	cacaaagatgccttttctaccg	RT-qPCR
21	mErdj4.AP1	*Erdj4*	ttaaacttttcagcttaatgacgtg	RT-qPCR
22	mGrp94.SP1	*Grp94*	acacactaggtcgtggaaca	RT-qPCR
23	mGrp94.AP1	*Grp94*	gtctctgtcttgctactccaca	RT-qPCR
24	mGrp78.SP1	*Grp78*	ctgaggcgtatttgggaaag	RT-qPCR
25	mGrp78.Ap1	*Grp78*	tcatgacattcagtccagcaa	RT-qPCR
26	mHerp.SP1	*Herp*	acctgagccgagtctaccc	RT-qPCR
27	mHerp.AP1	*Herp*	aacagcagcttcccagaataaa	RT-qPCR
28	m18s.SP1	18*s*	gtaacccgttgaaccccatt	RT-qPCR
29	m18s.AP1	*18s*	ccatccaatcggtagtagcg	RT-qPCR
30	rPerk-sgRNA.SP1	*Perk*	catcggatacggcatttggc	sgRNA
31	rPerk-sgRNA.AP1	*Perk*	gccaaatgccgtatccgatg	sgRNA
32	rPerk-sgRNA.SP2	*Perk*	agatggacgaattgccgcac	sgRNA
33	rPerk-sgRNA.AP2	*Perk*	gtgcggcaattcgtccatct	sgRNA
34	rPerk-sgRNA.SP3	*Perk*	catacgggctcagtgcatat	sgRNA
35	rPerk-sgRNA.AP3	*Perk*	atatgcactgagcccgtatg	sgRNA

### Immunoblot analysis

Cells were washed in ice-cold PBS and lysed in TNT buffer (50 mM Tris [pH 7.5], 150 mM NaCl, 10% glycerol and 1% Triton X-100) with protease inhibitor cocktail (Nacalai Tesque), phosphatase inhibitor cocktail (Biotool) and 10 μM MG132 (Enzo Life). Immunoblot analysis was performed as previously described using Blocking One (Nacalai Tesque) or Blocking One-P (Nacalai Tesque) and WesternSure ECL Substrate (Li-Cor Biosciences). Protein was visualised using Ez-Capture II (ATTO Corp), and the band intensities were quantified using Image Studio software (Li-Cor Biosciences). The antibodies for immunoblotting were as follows: anti-phospho-Ser51-eIF2α (Cell Signaling Technology), anti-phospho-Ser51-Perk (Cell Signaling Technology), anti-ATF4 (Sigma Aldrich), anti-KDEL (Abcam), anti-β-actin (Abcam) and anti-COL2A1 (Rockland). Anti-Chop and anti-Xbp1 were kindly gifted by Dr. David Ron (Cambridge Institute for Medical Research).

### Immunohistochemistry

To detect extracellular COL2A1, immunohistochemistry was performed using standard protocols. Briefly, 16.5 or 18.5 dpc embryonic mouse tibiae were fixed in 4% paraformaldehyde and decalcified in Morse’s solution. After defatting using 70% ethanol extraction, decalcified tissues were embedded in paraffin and sectioned into 5-μm slices. Deparaffinized sections were stained with rabbit anti-COL2A1 (Rockland), and the primary antibody was visualised using anti-rabbit Alexa Fluor 488 (Molecular Probes). Fluorescence detection was performed using an FV10i laser-scanning confocal microscope (Olympus), and fluorescence intensities were measured using ImageJ. To detect intracellular COL2A1 expression, mouse primary chondrocytes were treated with 2 μM GSK2606414, fixed in 70% ethanol, and stained with rabbit anti-COL2A1 and mouse anti-GM130 (BD Biosciences) at 4 °C for 12 h. Primary antibody was visualised using anti-mouse Alexa Fluor 488 and anti-rabbit Alexa Fluor 568 (Molecular Probes), and then, cells were stained with Hoechest 33258 (Invitrogen). Fluorescence detection was performed using a DMI 6000B fluorescence microscope (Leica) or FV10i laser-scanning confocal microscope, and fluorescence intensities were measured using ImageJ or Fluoview acquisition software (Olympus)^49^. To detect cell differentiation, ATDC5 cells were stained with alcian blue as previously described^45^.

### ATF4 activation analysis

To monitor ATF4 activation, ATDC5 cells were transduced with lentiviral vectors encoding an EGFP reporter gene driven by 25 repeats of the AARE or control EGFP lentiviral vectors. The green fluorescence of EGFP was visualised using a DMI 6000B fluorescence microscope, and fluorescence intensities were measured using ImageJ.

### Collagen secretion analysis

ER-to-Golgi protein trafficking was measured using a previously published protocol with modifications^29,30^. Briefly, mouse primary chondrocytes were synchronised by inhibiting the exit of cargoes from the ER by shifting the temperature to 40 °C for 3 h Then, the cargoes were permitted to synchronously exit from the ER by adjusting the temperature to 32 °C combined with a 100 μg/ml CHX (Nacalai Tesque) chase in the presence of 50 μg/ml ascorbic acid (Sigma). At each CHX chase point, cells were fixed and subjected to immunohistochemistry. To measure extracellularly secreted collagen by immunoblot analysis, RCS cells were incubated in DMEM supplemented with 2% FBS for 4 h. Clarified culture supernatants from RCS cells were precipitated in the presence of 10% trichloroacetic acid. After washing in acetone, the precipitates were dissolved in urea loading buffer and then subjected to SDS-PAGE for immunoblot analysis. To measure extracellularly secreted collagen using ELISA, RCS cells or primary chondrocytes were incubated in Opti-MEM (Gibco) for 6 h or 12 h, and clarified culture supernatants from cells were directly subjected to secreted COL2A1 measurement using type II collagen detection ELISA kit (Chondrex).

### HMW detergent-resistant complex detection

To estimate misfolded protein content in the ER, HMW detergent-resistant complex detection was performed using a previously published protocol with modifications^31^. Cultured cells were incubated for 5 min in ice-cold PBS containing 20 mM NEM and then collected by scraping in lysis buffer (0.5% Triton X-100, 20 mM HEPES [pH 7.5], 250 mM sucrose, 100 mM NaCl and 2.5 mM CaCl_2_) with protease inhibitors. Equal amounts of protein were adjusted to 0.8% SDS and then layered upon separation buffer (0.5% Triton X-100, 0.8% SDS, 20 mM HEPES [pH 7.5], and 20% glycerol) and centrifuged at 100,000 × *g* for 45 min. The resulting pellet was dissolved in urea loading buffer (9.6 M urea, 1.36% SDS, 40 mM Tris [pH 6.8], 12% glycerol, 100 mM DTT and 0.002% bromophenol blue), boiled for 5 min and then subjected to SDS-PAGE for immunoblot analysis.

### Proliferation assays

To evaluate chondrocyte proliferation in cartilage, pregnant females were injected at 16.5 or 18.5 dpc with 0.25 mg/g BrdU (TCI) 2 h before harvesting embryos. BrdU was detected using a BrdU staining kit (Abcam) based on the manufacturer’s protocol. To evaluate cultured RCS cells, cells were pulsed with 20 mM BrdU for 30 min, fixed with 70% ethanol and stained with mouse anti-BrdU antibody (Biolegend) at 4 °C for 12 h. The primary antibody was visualised using anti-mouse Alexa Fluor 488, and then, cells were stained with Hoechest 33258. Fluorescence was visualised using a DMI 6000B fluorescence microscope.

### Apoptosis detection assays

The ApopTag *in situ* apoptosis detection kit (Millipore) was used for TUNEL staining of mouse embryonic cartilage based on the manufacturer’s protocol, and TUNEL-positive cells were visualised using an Axioplan 2 fluorescence microscope (Zeiss). Apoptosis in the cultured chondrocytes was analysed using an ELISAPlus cell death detection kit (Roche Diagnostics).

### Statistical analysis

Unless otherwise specified, data shown in each figure are representative of at least three biological replicates and all data were expressed as means ± standard deviations and analysed using Student’s *t*-test. *P* < 0.05 was defined as the threshold of significance.

## Electronic supplementary material

Supplemental figures
