# Supplementary material for: PERK-mediated translational control is required for collagen secretion in chondrocytes
Source: Sci Rep. 2018 Jan 15;8:773. doi: 10.1038/s41598-017-19052-9 (PMC5768675; doi:10.1038/s41598-017-19052-9)
Supplement: Supplementary file 1 — Supplemental figures [file 41598_2017_19052_MOESM1_ESM.pdf]

PERK-mediated translational control is required for collagen secretion in chondrocytes

Satoshi Hisanaga1,4, Masato Miyake1,2,3, Shusuke Taniuchi1,2, Miho Oyadomari1,2, Masatoshi Morimoto1, Ryosuke Sato1, Jun Hirose5, Hiroshi Mizuta4 and Seiichi Oyadomari1,2,3*

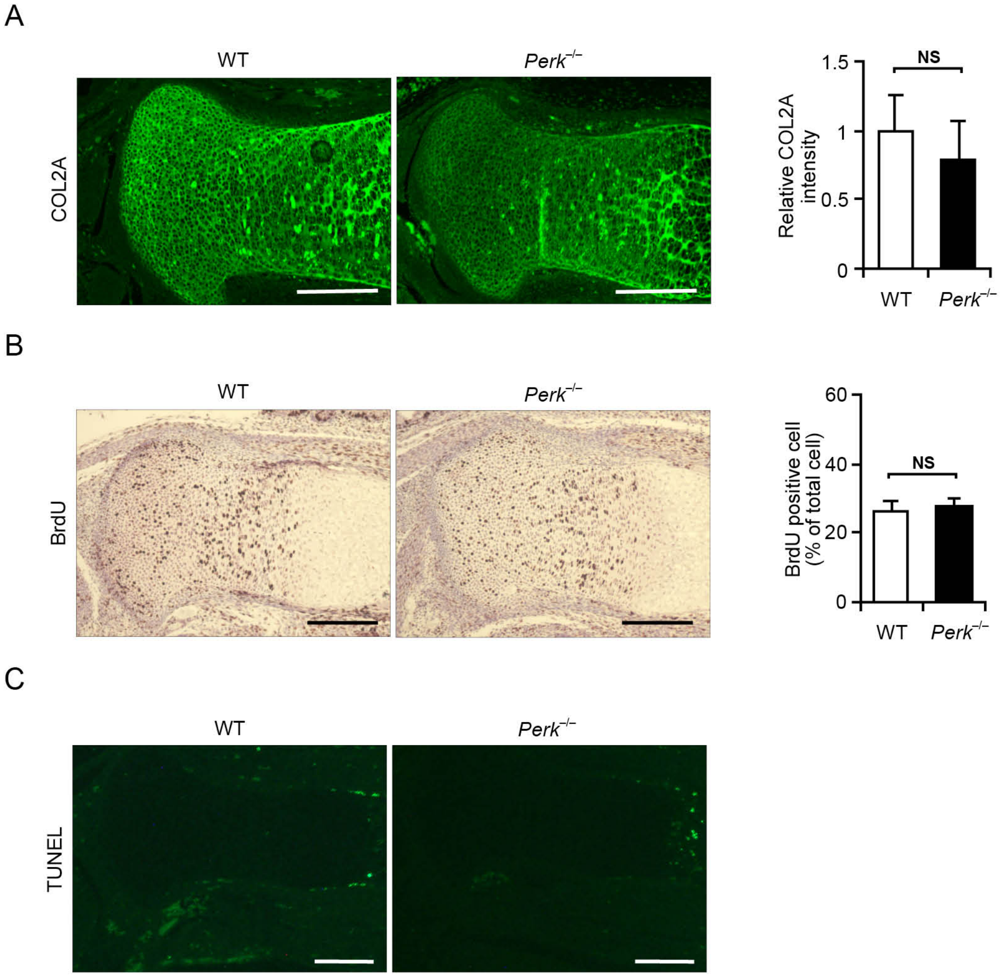

Supplemental figure 1

(A) Representative fluorescence micrographs of immunohistochemistry for COL2A1 of tibial sections from wild-type or Perk−/− mice at 16.5 dpc. Scale bar, 200 μm. Relative fluorescence intensity was presented as the mean fold change ± SD versus that of wild-type mice (n = 4 technical replicates, NS = not significant).

(B) Representative micrographs of immunohistochemistry for bromodeoxyuridine (BrdU) in tibial sections from wild-type or Perk−/− mice at 16.5 dpc. The sections were counterstained using hematoxylin. Scale bar, 200 μm. Ratio of BrdU-positive cells divided by the total number of cells was expressed as the mean ± SD (n = 4 technical replicates, NS = not significant).

(C) Representative micrographs of TUNEL staining of tibial sections from wild-type or Perk−/−mice at 16.5 dpc. Scale bar, 200 μm.

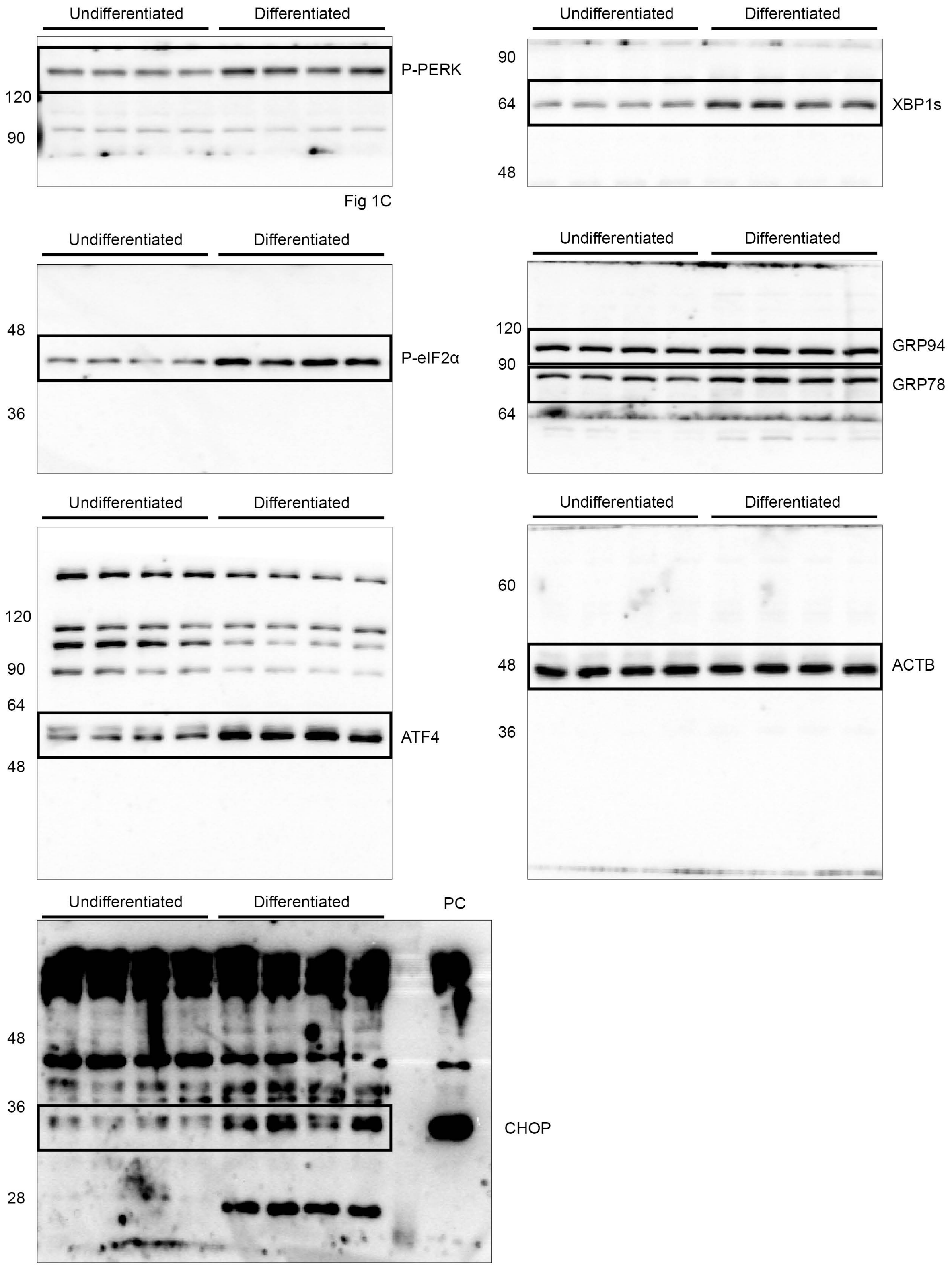

ATDC5 cell treated with 2 μg/ml tunicamycin was used as a positive control (PC).

Supplementary Figure 2. Uncropped images for immunoblots for Fig 1C.

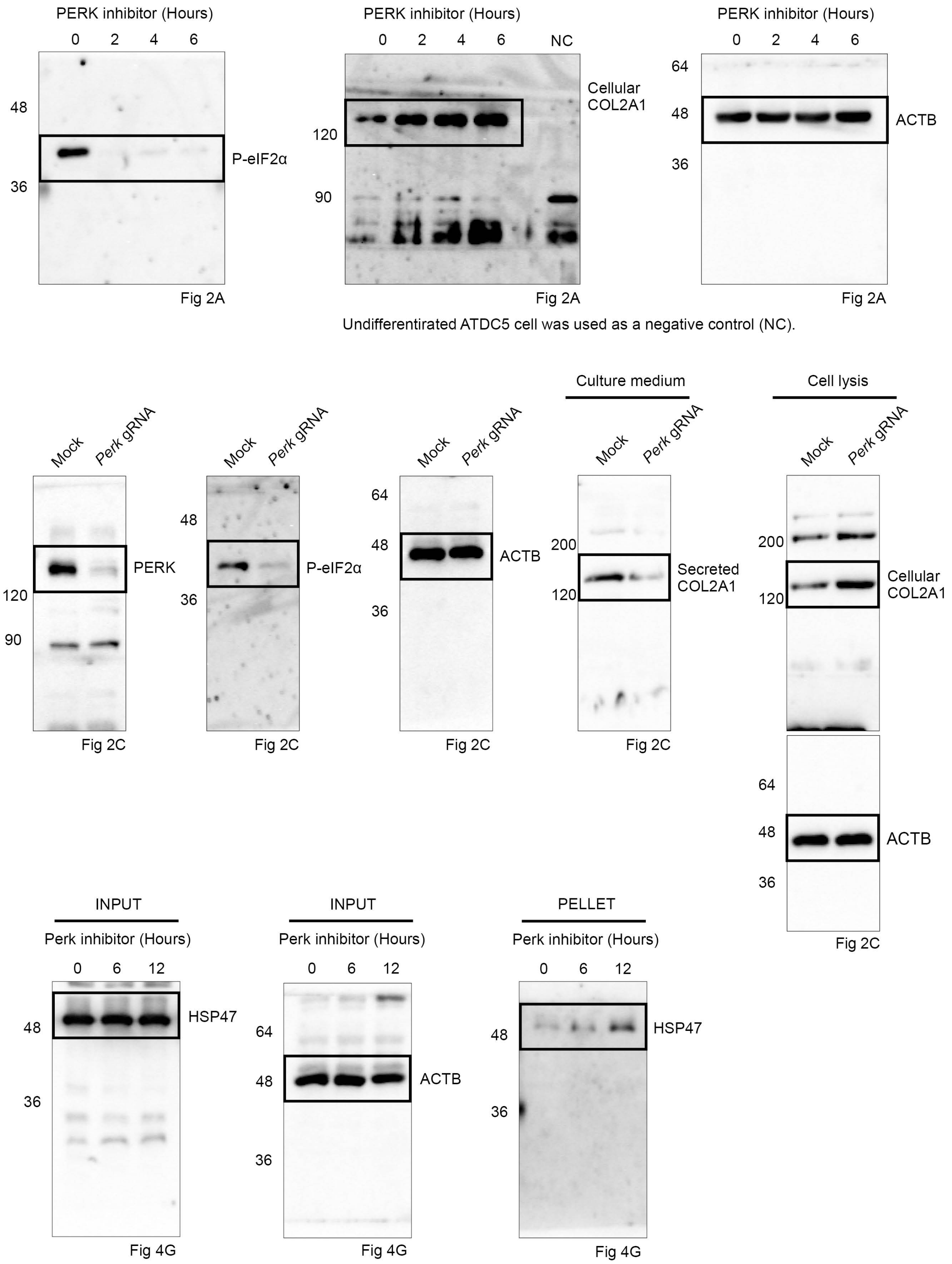

Supplementary Figure 3. Uncropped images for immunoblots for Fig 2A, 2C and 4G.
